# Inhibition and cognitive workload during deception about planned behavior: Investigating LPC, MFN, and PRP components

**DOI:** 10.3758/s13415-026-01430-4

**Published:** 2026-03-18

**Authors:** Vera Scheuble-Cabrera, Emely Voltz, André Beauducel

**Affiliations:** Institute of Psychology, University of Bonn, Kaiser-Karl-Ring 9, 53111 Bonn, Germany

**Keywords:** Deception, Medial frontal negativity, Late positive components, Theory of planned behavior, Machiavellianism, Response times

## Abstract

The present preregistered study investigated cognitive processes during deception about planned behavior. It extends prior research on deception about attitudes by addressing individual life plans as a more specific and immediate determinant of future behavior. By examining deception about individually planned behaviors, the present study establishes a link to previous forensic research focusing on deception concerning (criminal) behavior. Participants (*N* = 114) indicated whether they planned certain behaviors. Stimuli were selected individually for each participant. Participants responded more slowly and less accurately for deceptive than truthful responses. Medial frontal negativities (MFNs) were enlarged for deceptive compared to truthful responses, revealing greater response conflicts during lying. Decreased pre-response positivities (PRP) and late positive components (LPCs) for deceptive responses suggested that lies were accompanied by strategic monitoring and were cognitively more demanding, respectively. For all ERP components, the difference between deceptive and truthful responses was greater for planned than for unplanned behavior, revealing that lying about planned behavior was cognitively more challenging. Practice, i.e., repeating the task, did not affect the difference in ERPs between deceptive and truthful responses, indicating that deception did not become less cognitively demanding. The patterns of MFN and LPCs were stable across individual differences in Machiavellianism. The MFN, PRP, and LPC components appear to be promising markers of the additional executive processes involved in lying about individual future life plans. These findings provide a foundation for investigating ERP components during deception also regarding other nonforensic, everyday topics.

**Supplementary Information:**

The online version contains supplementary material available at 10.3758/s13415-026-01430-4.

## Introduction

Deception is an everyday phenomenon (Ennis et al., 2008; Kashy & DePaulo, 1996). In a diary study by DePaulo et al. (1996), the content of lies was assessed. They found that plans are one of the main topics individuals lie about. Planning to meet friends, doing one’s taxes, studying more, searching for new jobs, eating healthier, donating money, or taking a flight are just some plans people might lie about. Plans for one’s own future life, or in other words individual goals, are important in everyday life, because they shape the behavior of people, have to be kept in mind to be achieved, and are crucial for a meaningful life (Emmons, 2003). To better understand the cognitive processes underlying lies and their corresponding neural correlates, it is crucial to study lying about more types of stimuli. In most ERP studies about deception, participants have to conceal knowledge of an item previously seen during a mock crime. To the best of our knowledge, deception concerning individually planned behavior of everyday life (e.g., having children, graduating, learning a musical instrument) has not yet been investigated. To address this research gap, the present study investigated the cognitive processes underlying such lies in a controlled laboratory setting by analyzing behavioral and ERP components.

When investigating planned behavior, it is essential to introduce the Theory of Planned Behavior (TPB), which provides an important theoretical framework for understanding this construct (Ajzen, 1985). For many, if not all, behaviors, the plan to perform them constitutes a crucial step prior to their execution (Ajzen, 1985). Even for highly habitualized behavior, such as sending a message, an explicit or implicit plan guides the execution (Ajzen, 1985). Based on Ajzen (1985, p. 24), planned behavior can be defined as “a behavior-goal unit; and the intention constitutes a plan of action in pursuit of the behavioral goal.” The plan is described in the TPB as the intention to perform the behavior and is the immediate determinant of the action (Ajzen, 1991). Furthermore, according to Ajzen (1991, p. 181), “[i]ntentions are assumed to capture the motivational factors that influence a behavior; they are indications of how hard people are willing to try, of how much of an effort they are planning to exert, in order to perform the behavior.” In this line, we conceptualize *planned behavior* as behavior that is *wanted* and *unplanned behavior* as *unwanted behavior*. It has been found that attitudes are rather poor predictors of behavior in specific situations (Ajzen, 1991). Ajzen (1991) explained this result by referring to the level of aggregation of attitudes, which is possibly too general to predict specific behavior. To better predict behavioral execution, a more specific predictor is required (Ajzen, 1991): the plan—or, in other words, the intention—to perform the behavior. This plan is itself influenced by the attitude toward the behavior, but in contrast to attitudes, serves as a direct predictor of behavior (Ajzen, 1991; Ajzen & Kruglanski, 2019). Likewise, motivational theories formulate planning as a phase immediately preceding an action (Gollwitzer, 1996). Whereas individuals have many wishes, only some of them are further pursued. Plans are specified in implementation intentions. According to Gollwitzer (1996, p. 292), the planning phase starts with the formulation “I intend to achieve X!” At this stage, individuals are more determined and committed to realizing their behavioral intentions. Because high commitment of individuals to their own plans can be assumed, one might expect substantial ERP effects when lying about their individual plans. Individual plans are, of course, stored in memory. This is not the result of memorizing them immediately before the task, because they are represented in memory for a longer period of time. Moreover, the process of response inhibition during deception can be expected to be strong for individual plans, given individuals’ commitment to their own plans. The combined effect of long-term memory and high individual commitment can also be found in concealed information tests (CITs) based on stimuli from true crimes. In this sense, the investigation of individual plans is similar to previous deception research based on true crimes. However, the main difference is that CITs employed in forensic settings typically refer to known stimuli related to the critical behavior, whereas in the present study, verbal expressions of individual plans are investigated. Therefore, the present study can help to clarify whether the combined effect of long-term memory and commitment, which are relevant in forensic, stimulus-based CITs, can be generalized to nonforensic, verbally coded individual plans.

After introducing the content of deception examined in the present study, the following section outlines the theoretical framework of deception. According to Vrij (2008, p. 15) deception can be defined as “a successful or unsuccessful attempt without forewarning, to create, in another a belief which the communicator considers to be untrue.” Deception relies on executive processes: The truth has to be inhibited, conflicts between the truth and lie occur, and a new consistent response has to be given (Johnson, 2014; Sip et al., 2008; Walczyk et al., 2003). Prior deception studies found that the additional cognitive demands of lying manifest behaviorally as less accurate and slower responses relative to truthful responding (Johnson et al., 2003, 2008; Scheuble et al., 2021; Scheuble & Beauducel, 2020a, 2020b; Suchotzki et al., 2015, for a review, see Suchotzki et al., 2017). Slower responses for deception have been primarily linked to response inhibition, task switching, and an increased working memory load, given that lying requires maintaining two competing responses—the truth and the lie (Suchotzki et al., 2017). Furthermore, cognitively more effortful processes for lies can be made apparent by ERPs. A prominent ERP in deception studies is the late positive component (LPC), also named P300 (Johnson et al., 2003, 2004, 2005, 2008; Meijer et al., 2007; Polich, 2007). In the following, we apply the term LPC, since the LPC and P300 have not been defined by invariant features and the terms have been used interchangeably (Leue & Beauducel, 2019; Polich, 2007, p. 2128). The LPC has been related to attentional allocation (Polich & Kok, 1995; Pritchard, 1981). Enlarged LPC amplitudes occurred for salient or meaningful stimuli, such as oddballs—that is, infrequently presented stimuli among a series of frequent ones (Hruby & Marsalek, 2003; Johnson, 1986; Polich & Criado, 2006). At the same time, the LPC is sensitive to information transmission (Johnson, 1986). When the attention is drawn away from a stimulus—for example by an additional task—its processing is hampered, and decreased LPC amplitudes were found (Johnson, 1986, 1988). Accordingly, decreased LPC amplitudes were also found with increasing task difficulty, like in dual tasks (Beauducel et al., 2006; Miller et al., 2011; Palmer et al., 1994). In most deception studies, people have to conceal that they recognize a certain memorized probe stimulus in a series of other irrelevant items during a CIT. The probe item is presented less frequently than the other group of irrelevant items. When individuals recognize the probe item and conceal their knowledge of it, it appears more salient to them, as indicated by more positive LPC amplitudes in previous CIT studies (Leue & Beauducel, 2019). There are only a handful of studies in which deception was not analyzed in recognition tasks (Dong et al., 2010; Johnson et al., 2008; Meek et al., 2013; Pfister et al., 2014; Suchotzki et al., 2015; Scheuble & Beauducel, 2020a). In these tasks, in which participants have to lie as frequently as they give truthful responses, converse patterns of LPC amplitudes were found. Suppressed (i.e., less positive) LPC amplitudes occurred for deceptive than for truthful responses, indicating that lying was cognitively more strenuous. Lying can be considered an additional task, requiring more effort and therefore resulting in suppressed LPCs (Beauducel et al., 2006; Johnson et al., 2008). Whereas concealing knowledge for probe items in CITs is associated with higher stimulus salience and larger LPC amplitudes, we expect that deception about planned behavior requires additional executive processes, thereby inducing greater cognitive effort compared with truthful responses about planned behavior. Consequently, we expect LPC amplitudes to be suppressed for deceptive responses about planned behavior relative to truthful responses.

An ERP more rarely investigated in the context of deception is the medial frontal negativity (MFN). Originally, a negative deflection was observed at frontocentral electrodes following erroneous responses, referred to as the error-related negativity (ERN). Later, a similar deflection was found for correct responses in tasks requiring the co-activation of different responses (Bartholow et al., 2005; Gehring & Knight, 2000). In deception studies, a comparable negative deflection, occurring shortly after the response at frontocentral electrodes was observed and termed MFN or, in some studies, correct-response negativity (CRN; Johnson et al., 2004, 2005, 2008; Scheuble & Beauducel, 2020a; Suchotzki et al., 2015). The assumed source of the MFN is the anterior cingulate cortex (ACC; Gehring & Willoughby, 2004; Nieuwenhuis et al., 2004). The ACC and the MFN have been associated with response inhibition and monitoring of response conflicts (Botvinick, 2007; Johnson et al., 2008). In some deception studies, more negative MFN amplitudes were found for lies, a pattern that has been associated with greater response conflicts for deceptive than for truthful responses (Gibbons et al., 2018; Johnson et al., 2004, 2005, 2008; Leue et al., 2012; Scheuble & Beauducel, 2020a, 2020b; Scheuble et al., 2021).

In previous studies by Johnson et al., (2004, 2005, 2008) and in a replication of one of their studies (Scheuble & Beauducel, 2020a), a positive deflection occurred preceding a response, which was named pre-response positivity (PRP; Johnson et al., 2004). Attenuated PRP amplitudes were linked to strategic monitoring and “processing required to make intention-based responses” (Johnson, 2014, p. 254). Such cognitive processes are required when a long-term goal needs to be remembered and behavior has to be directed towards this goal. Johnson et al., (2004, 2005) compared PRP amplitudes for directed lies and self-generated lies. For directed lies, participants were instructed to lie the whole task. For self-generated lies, participants had a choice to lie or be truthful on every trial but should lie as frequently as give truthful responses. PRP amplitudes were attenuated for both types of lies but were more strongly reduced for self-generated lies. Less positive PRP amplitudes could also be found for lies about attitudes (Johnson et al., 2008; Scheuble & Beauducel, 2020a). Accordingly, it was concluded that lying about attitudes also involves strategic monitoring, akin to self-generated lies (Johnson et al., 2008). Strategic monitoring also seems to be relevant for responses about planned behavior. When an individual strongly pursues a plan, it is maintained in mind, and responses and behaviors must be aligned with it to ensure successful future implementation. Likewise, when lying about planned behavior, strategic monitoring and inhibition of the individual plans seem to be required, as individual plans are present in long-term memory, have real consequences, induce high commitment, and manifest themselves in everyday life.

Johnson et al. (2008) analyzed LPCs, MFNs, and PRPs for lies about attitudes. Suppressed LPC amplitudes for deceptive compared to truthful responses revealed increased cognitive demands for lies. Enlarged MFN amplitudes and suppressed PRP amplitudes for deceptive in comparison to truthful responses indicated greater response inhibition and strategic monitoring for lies. Furthermore, lying was cognitively more challenging and accompanied by greater response inhibition and strategic monitoring for positively than for negatively valued items. These findings were all replicated by Scheuble and Beauducel (2020a). Likewise, Johnson et al. (2008) reported greater differences in ERPs and response times for attitudes than for other self-referential stimuli and explained this result by the fact that attitudes guide everyday behavior and are important for individuals (Johnson, 2014). Even though plans are more closely related to people’s future behavior, they are, like attitudes, important to people, need to be kept in mind, and require that behavior be adapted to them in order to achieve them (Berkman, 2018; Gollwitzer & Brandstätter, 1997; Locke & Latham, 2002). Plans influence attention, effort, and persistence in goal-directed behavior (Locke & Latham, 2002). Plans should therefore be easily accessible from long-term memory, and stating something untrue about them should contrast with their easily accessible and personally meaningful true content and thus be accompanied by greater response conflicts and higher cognitive demands. Therefore, similar patterns in MFNs, LPCs, PRPs, and response times between deceptive and truthful responses can be expected for plans, as previously observed in the context of attitudes.

Very few ERP studies have investigated deception in the context of planned behavior, and, to the best of our knowledge, no previous research has examined it in a nonforensic context. A study by Meixner and Rosenfeld (2011) analyzed LPC components in the context of a planned terrorist attack. However, they did not analyze lies about the plan itself but about the recognition of items related to the terrorist attack during a CIT, in which typically enlarged LPC amplitudes are found for concealed knowledge. In line with this, LPC amplitudes were larger for the probe item associated with the terrorist attack, for which participants concealed knowledge, than for irrelevant items that were unknown to them. Accordingly, the probe item, for which participants had to conceal knowledge, appeared more salient than the other irrelevant unknown items. In a study by Suchotzki et al. (2015), participants planned a theft. During a Sheffield lie test, LPC components were suppressed for deceptive compared with truthful responses, revealing increased cognitive workload during lies. However, unexpectedly, MFN amplitudes were more negative for truthful than for deceptive responses. The distinct patterns of LPC components in different lie tasks and the unexpected MFN finding reported by Suchotzki et al. (2015) underscore the need for additional research in this area.

Whereas mock-crime CITs refer to memory of stimuli that mostly occurred in the context of already performed behavior, the investigation of deception effects that are not primarily due to the recognition of previously memorized stimuli was not related to concrete behavior but to attitudes. There is therefore a gap between CIT studies, which are typically related to the recognition of stimuli associated with behavior, and non-CIT studies, which are not related to recognition of stimuli but, at the same time, are not directly linked to behavior. It is therefore not clear whether the different results from CIT and deception tasks about attitudes were related to the fact that attitudes are less closely linked to concrete behavior than the stimuli in a mock-crime CIT. The purpose of the present study was to close this gap. The research question is whether deception effects found for attitudes and memory (Johnson et al., 2003, 2004, 2005, 2008; Scheuble & Beauducel, 2020a), which do not necessarily imply substantial personal involvement at the level of individual activity, can be generalized to individually planned behavior. However, according to the TPB (Ajzen, 1991), attitudes towards behavior can find expression in behavior only if the respective behavior is under volitional control. Therefore, the present investigation focused on planned behavior. The present study extends existing knowledge by addressing deception about future planned events that are likely, yet not guaranteed, to occur. We investigate deception about a rarely studied temporal dimension—the planned future—aside from forensic studies by Rosenfeld and Meixner (2011) and Suchotzki et al. (2015). Because this is one of the first studies investigating deception about planned behavior in a nonforensic context, it was conducted in a controlled laboratory setting. This approach was chosen to enable better comparison of similarities and discrepancies with findings from previous laboratory deception studies on attitudes and those with a forensic context.

When studying deception, not only general processes but also possible moderating effects—such as those stemming from individual differences or task composition—need to be considered. A personality construct that seems relevant when investigating deception about planned behavior is Machiavellianism. The importance of considering individual differences was already emphasized in the TPB (Ajzen, 1991). Individuals high in Machiavellianism have fewer difficulties disregarding emotionally irrelevant information and staying focused on their goals (Geis et al., 1970). Consequently, strategic planning represents a core characteristic of Machiavellianism (Jones & Paulhus, 2014). Moreover, Machiavellianism has been related to deception (Jonason et al., 2014; Jones & Paulhus, 2009; Kashy & DePaulo, 1996; Yarbrough & Hart, 2025). Machiavellianism is characterized by manipulative and strategic tactics, such as deception, a callous affect, cynical worldview, and a lack of conventional morality (Christie & Geis, 1970; Jones & Paulhus, 2014). Individuals scoring higher on Machiavellianism lie more frequently and indicate that lying is cognitively less effortful (Azizli et al., 2016; Gozna et al., 2001; Murphy, 2012). They evaluate their lying abilities as higher and report feeling less guilt when lying compared with individuals scoring lower on Machiavellianism (Gozna et al., 2001; Murphy, 2012; Wissing & Reinhard, 2019). Only Machiavellianism was a significant predictor of high-stakes deception over and above the other Dark Triad traits in Azizli et al. (2016). Likewise, it has been found that individuals higher in Machiavellianism tell more white lies (Jonason et al., 2014). Yet, the results of deception studies regarding the association between Machiavellianism and ERPs are mixed: Panasiti et al. (2014) found a more suppressed Bereitschaftspotential for participants scoring lower on Machiavellianism. Patterns of the Bereitschaftspotential were linked to moral conflicts and moral dilemmas. Accordingly, individuals scoring higher on Machiavellianism experienced fewer moral conflicts during deception. Scheuble and Beauducel (2020b) reported that women scoring higher on Machiavellianism experienced less response conflict during prosocial lies: Machiavellianism moderated the difference in MFN amplitudes between deceptive and truthful responses for women who witnessed and lied about a social conflict. In the same study, no significant moderation effect of Machiavellianism was found on LPC amplitudes. In a study based on the CIT, high psychopathic individuals did not differ from low psychopathic individuals in their LPC amplitudes (Miller & Rosenfeld, 2004). Furthermore, Scheuble and Beauducel (2020a) found no moderation effect on LPC and MFN amplitudes in a deception task about attitudes. In sum, Machiavellianism is linked to both deception and planning, and it thus appears relevant to consider its potential moderating role when studying deception about planned behavior.

Regarding the task composition of EEG studies, it must be considered that physiological responses to a single trial are influenced not only by the process of interest (such as responding deceptively) but also by background noise (Luck, 2014). Therefore, in ERP studies, the trials of the task are repeated numerous times, and EEG activity is averaged for the event of interest. Increasing the number of trials can improve the signal-to-noise ratio. Accordingly, to ensure that enough trials are applied to study the cognitive processes underlying deception in a noise-free manner, it seems advisable to use as many trials as possible. To this end, we repeated the parts of the deceptive and truthful task block. Yet, when increasing the number of trials, potential practice effects have to be taken into account. Johnson et al. (2005) reported practice effects in the MFN, LPC, and PRP amplitudes when comparing truthful and deceptive responses. In a repeated truthful task block, LPC amplitudes were increased, MFN amplitudes were decreased, and PRP amplitudes tended to be decreased. In contrast, no such changes were observed in any of the described ERP components when the deceptive task block was repeated. Other studies on the effects of practice on ERP components during deception tasks are scarce. Larson et al. (2010) found that ERN amplitudes were stable across repetitions of a flanker task. In another study, decreases in LPC amplitudes were found over task blocks in which participants had to discriminate between two equiprobable stimuli (Ravden & Polich, 1999). Because practice effects on ERP amplitudes in deception tasks cannot be ruled out, it appears necessary to include a manipulation check when repeating task blocks to increase the signal-to-noise ratio. Accordingly, we accounted for the effects of task repetition in our statistical analyses. Knowing whether such repetitions influence ERP components is important for the methodology of future studies.

### Aims and hypotheses

To sum up, we investigated the cognitive processes during deception about planned behavior. Thereby, we examined whether previous results found for deception about attitudes can be generalized to deception about planned behavior. By studying planned behavior, the proximity to concrete behavior and the commitment to it should be maximized, with more commitment possibly implying intense inhibition effects when lying. At the same time, the effects of recognition memory should be minimized. The latter can be assumed because the representation of individual plans in long-term memory should be well established. All hypotheses regarding MFN and LPC amplitudes were preregistered (https://osf.io/6syqj/?view_only=cde80f6b7f494791ade8725444da6fb4). Because lying should be accompanied by more conflicts, we expected larger (i.e., more negative) MFN amplitudes for deceptive compared with truthful responses about individual plans (pre-registered hypothesis 1). Furthermore, as lying should be cognitively more demanding than truth-telling—comparable to performing an additional task—we expected suppressed LPC amplitudes for deceptive responses about individual plans (pre-registered hypothesis 2). Moreover, we explore whether the difference in both ERP amplitudes between deceptive and truthful responses is more pronounced for planned behavior in comparison to unplanned behavior. Given that Machiavellianism is characterized by both deception and strategic thinking, but previous studies have reported mixed findings regarding its moderating role in deception, we explored whether Machiavellianism moderates the effect of deception on the ERP amplitudes. The analyses regarding the PRP amplitude were not preregistered. Nevertheless, given that strategic monitoring appears to be relevant for deception about plans, the PRP amplitude was analyzed post hoc as suggested by reviewers to determine whether similar patterns can be found to those reported in previous studies on deception about attitudes (Johnson et al., 2008; Scheuble & Beauducel, 2020a). Analyzing the PRP also appeared to be relevant, as it is a newly identified component that has so far only been reported for self-generated lies and lies about attitudes. Observing this component during deception about planned behavior would corroborate its theoretical and practical importance. Methodical analysis steps for the analysis of the PRP were the same as in former studies for lies about attitudes (Johnson et al., 2008; Scheuble & Beauducel, 2020a).

## Method

### Participants

The sample size required to obtain high statistical power was calculated with G*Power 3.1.9.7 (Faul et al., 2009). In a similar deception study by Scheuble and Beauducel (2020a), effect sizes of at least *d* = 0.54 were reported for differences in MFN as well as LPC amplitudes. To reach a statistical power of .95 for a repeated-measures test at an alpha level of *p* <.05, two-tailed, a sample size of 47 participants is required. As large sample sizes have been recommended under the background of the replication crisis, and we additionally explored moderation effects of Machiavellianism, our pre-registered set and accomplished goal was to recruit 120 participants (https://osf.io/6syqj/?view_only=cde80f6b7f494791ade8725444da6fb4). Participants with less than 20 artifact-free epochs and correct trials for the main categories planned vs. unplanned behavior for truthful/deceptive responses were excluded (*n* = 3). To ensure that participants did not swap the meanings of the response buttons in the deception task, we employed two manipulation checks and excluded participants when necessary. As in previous studies using a similar deception task (Johnson et al., 2003, 2005, 2008; Scheuble & Beauducel, 2020a), the deception task included catch trials. In these catch trials, participants had to truthfully indicate the button assignments (see section *Deception Task*). If participants did not swap the meaning of the response buttons, response times were expected to be longer for trials requiring a deceptive response to the items than for catch trials. However, if participants did swap the meanings of the response buttons, response times were expected to be longer for catch trials, and these participants were excluded. Additionally, at the end of the computer task, participants were asked whether they had used any strategies during the deception task, which those were, and whether they had switched response buttons. Participants who reported having switched response buttons were also excluded. In total, three additional participants were excluded, as the manipulation checks indicated that they had likely reversed the button assignments during the deception task. This resulted in a final sample of 114 participants (59 males; age: mean [*M*] = 23.50, standard deviation [*SD*] = 3.98, range = 18–38 years). Participants were right-handed, native German speakers, had normal or corrected-to-normal vision, and participated voluntarily. None of them had any neurological disorders. Psychology students (49%) got course credit for participating. The study was in accordance with the revised Helsinki declaration (1991), approved by the local ethics board of the Institute of Psychology from the University of Bonn, Germany (date: October 1, 2022; #22–10-04), and all participants signed informed consent.

### Measures

Two Machiavellianism scales were applied to control for different conceptions of the construct (Henning & Six, 1977; Jones & Paulhus, 2014). The two scales correlated significantly with each other, *r* =.62 (*p* <.001). Additionally, a joint score was formed as an equally weighted aggregate of both scales (hereafter referred to as joint scale). Each of the three Machiavellianism scores was analyzed in separate ANOVAs (see *Statistical Analyses* section). Cronbach’s alpha was α = .81 for the scale by Henning and Six (1977) and α = .72 for the scale by Jones and Paulhus (2014). In the questionnaire by Henning and Six (1977), participants had an overall mean of 2.56 (*SD* = 0.60) on the six-point Likert scale. In the questionnaire by Jones and Paulhus (2014), participants had an overall mean of 2.86 (*SD* = 0.58) on the five-point Likert scale. The questionnaire on plans consisted of 168 items, including major to minor life events (e.g., marriage, graduating, having children, going on a sabbatical, learning a musical instrument; see supplement). Participants were asked on a scale from one to seven to what extent they plan to do the event (1 = absolutely unwanted; 7 = absolutely wanted) and how likely they hold on to their plan (1 = absolutely unlikely; 7 = absolutely likely). Some of the items were based on plans, which proved to be important for people in previous studies or implemented in theories (Ajzen, 1991; Emmons, 2003; Pöhlmann & Brunstein, 1997). We ensured during an online pilot study that events from different life areas (work, religion, culture, sports, politics, home, and family) and related to different modalities (affective, material, cognitive; Sagie & Elizur, 1996) were presented. In the pilot study, 13 psychology students completed the questionnaire about plans and categorized the items based on the above-mentioned life areas and modalities. For the deception task of the main study, we selected for each participant 17–20 items with the highest wanted (rated as 7 or 6) as well as unwanted ratings (rated as 1 or 2). When this resulted in more than 20 items for a participant, we first selected items with the highest wanted/unwanted ratings and secondly those that were also highly held onto (Johnson et al., 2008; Scheuble & Beauducel, 2020a). When still more than 20 items were available, we selected the shorter items.

### Deception task

**Fig. 1:**
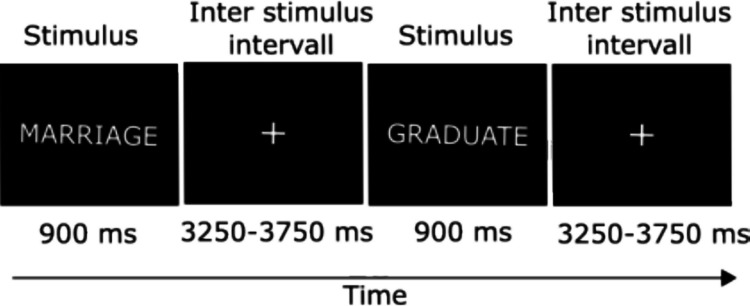
Sequence of two trials of the deception task

The deception task was completed during EEG recording on a computer in a sound-attenuated, electrically shielded, well-lit room. Participants saw the chosen items from the questionnaire about their plans. They were instructed to indicate whether they plan to perform each behavior by pressing either the left or right of two buttons. The task consisted of a deceptive condition block and a truthful condition block (Dionisio et al., 2001; Hu et al., 2011, 2012; Johnson et al., 2008; Scheuble & Beauducel, 2020a, 2020b), which were subsequently repeated. Before the deceptive condition blocks, participants were instructed to lie about their plans, whereas during the truthful condition blocks, they were instructed to indicate their plans truthfully. The assignment of the response buttons and the order of the condition blocks (first truthful and afterward deceptive condition block or vice versa) were counterbalanced across participants. The 17–20 chosen items for the planned and unplanned behavior were presented twice per condition block. Each condition block comprised 16 catch trials, during which the button assignments (‘WANTED’ [‘GEWOLLT’] and ‘UNWANTED’ [‘UNGEWOLLT’]) were displayed on screen. Participants were instructed to respond truthfully to these prompts in both the truthful and deceptive condition blocks. For instance, they pressed the button that stood for “I want to/plan to do the event” when “WANTED” appeared. Catch trials should ensure that participants did not swap the meaning of the button assignments to simplify the task in the deceptive condition block (Johnson et al., 2008; Scheuble & Beauducel, 2020a, 2020b). One trial (Fig. 1) consisted of the stimulus word (900 ms) and an intertrial interval (ITI) involving a fixation cross (pseudo-randomized duration: 3,250–3,750 ms). Participants could respond during the presentation of the word or the subsequent ITI. The stimuli were identical across both the deceptive and truthful condition blocks; the only difference between them was the instruction to respond either deceptively or truthfully. For the catch trials, participants were required to truthfully indicate the meaning of the response buttons in both condition blocks. However, as in real-life deception of individual plans, participants therefore had to keep the true meaning of their responses in mind and had to inhibit their true plans.

### Procedure

Participants completed the questionnaire about their individual plans around 3 days before their EEG examination (*M* = 2.79, *SD* = 0.92). During the EEG examination, participants sat about 95 cm away from a 19″ flat screen. The deception task was presented with Presentation V20.1 (Neurobehavioral Systems). To ensure that participants understood the task, they completed an exercise before the first deceptive and truthful condition block. The trial sequence was the same as in the main task. The items “COMPLETING QUESTIONNAIRES” and “KISSING A FISH” were presented. All participants planned to complete questionnaires and did not plan to kiss a fish. As in the main task, the exercises involved catch trials.

### EEG recording and quantification

**Fig. 2:**
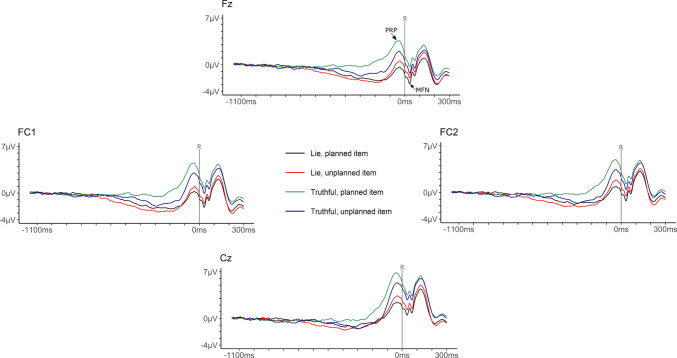
Response-locked grand averages of fronto-central electrodes for lies as well as truthful responses for planned behavior and unplanned behavior. Epochs spanned from 1,150 ms before until 300 ms after the response. One tick at the x-axis stands for 100 ms and one tick at the y-axis for 1 µV

The EEG was recorded by an ActiveTwo Biosemi system (Biosemi, Amsterdam, Netherlands). Sixty-four Ag/AgCl scalp electrodes were placed according to the extended 10/20 system (Jasper, 1958). The electrooculogram (EOG) was recorded with two horizontal electrodes at the epicanthi of both eyes and one vertical electrode placed approximately 1 cm below the middle of the right eye. As per Biosemi design, the Common Mode Sense active electrode and the Driven Right Leg passive electrode served as ground electrodes. Signals were digitized using ActiView software (BioSemi) with a sampling rate of 512 Hz. Electrode offsets were kept below 30 mV to avoid bad contact between electrodes and the scalp (Smith, 2007, p. 51). Offline analyses were performed with EEGlab (version 2022.1). Data were re-referenced to averaged pre-auricular electrodes and filtered, applying a 0.01–35 Hz band-pass (Johnson et al., 2008; Scheuble & Beauducel, 2020a). Independent component analysis served to correct ocular artifacts (ICA, with infomax decomposition). Data were segmented into epochs ranging from 1,150 ms before until 300 ms after the response (Johnson et al., 2008; Scheuble & Beauducel, 2020a, 2020b). As preregistered the baseline was set to −1,150 to −1,000 ms (Johnson et al., 2008; Scheuble & Beauducel, 2020a). The time window around the baseline showed negligible electrophysiological activity (Fig. 2). MFN and LPC components were quantified as mean amplitudes. Medial frontal negativity was measured between 10 and 80 ms at Fz, FC1, FC2, and Cz (Johnson et al., 2008; Scheuble & Beauducel, 2020a). The PRP amplitudes were measured between −100 and 0 ms at the same electrodes, i.e., Fz, FC1, FC2, and Cz (Johnson et al., 2008; Scheuble & Beauducel, 2020a). The LPC was measured between −100 and +100 ms at P3, Pz, P4, CP1, and CP2 (Johnson et al., 2008; Scheuble & Beauducel, 2020a).

### Statistical analysis

Statistical analyses were conducted with IBM SPSS (Version 28). Percentage of correct responses (responding as instructed) and response times served as behavioral data. Behavioral data and ERP components were analyzed by repeated-measures ANOVAs. Only correct trials were analyzed for ERPs and response times. A Response factor (truthful vs. deceptive responses), Repetition (repetition of truthful and deceptive condition blocks), and Plan (planned vs. unplanned behaviors) served as within-subject factors. For ERP analyses, the additional within-subject factor Electrode Position was entered. Separate repeated-measures ANCOVAs were conducted, analyzing moderating effects of Machiavellianism by including the mean-centered Machiavellianism score as a covariate and Response × Machiavellianism as an interaction term. To further explore potential interactions with Machiavellianism, we performed median splits for each Machiavellianism measure and conducted additional ANOVAs with Machiavellianism as a between-subjects factor. Effects of violations of the sphericity assumption were corrected by means of Greenhouse–Geisser, and the corresponding ε values are reported. Partial eta^2^ is reported as an effect size. Only two-tailed levels of statistical significance are reported.

## Results

### Ratings for plans

Participants rated the chosen planned behaviors as significantly more wanted (*M* = 6.84, *SE* = 0.24) than the chosen unplanned behaviors (*M* = 1.14, *SE* = 0.22, *t*(112) = 161.48, *p* <.001, *d* = 0.41) on the 7-point Likert scale (1 = absolutely unwanted; 7 = absolutely wanted). They indicated a significantly higher likelihood of maintaining their intentions regarding planned behaviors (*M* = 6.66, *SE* = 0.42) compared with unplanned behaviors (*M* = 5.82, *SE* = 0.19, *t*(112) = 4.36, *p* <.001, *d* = 0.38; Likert scale from 1 = absolutely unlikely to 7 = absolutely likely).

### Behavioral data

Correct responses were significantly more frequent in truthful (*M* = 96.04%, *SE* = 0.28) than deceptive condition blocks (*M* = 91.30%, *SE* = 0.54), *F*(1, 113) = 114.99, *p* <.001, η_*p*_^2^ =.50. Likewise, correct responses were significantly more common for planned behaviors (*M* = 94.64%, *SE* = 0.42) than for unplanned behaviors (*M* = 92.70%, *SE* = 0.48), *F*(1, 113) = 14.13, *p* <.001, η_*p*_^2^ =.11. Correct responses were also significantly more frequent in the repeated block (*M* = 93.98%, *SE* = 0.37) than in the first block of the task (*M* = 93.37%, *SE* = 0.42), *F*(1, 113) = 4.60, *p* <.05, η_*p*_^2^ =.04. For the percentage of correct responses, a significant Response × Repetition interaction occurred (Table 1), *F*(1, 113) = 11.18, *p* <.01, η_*p*_^2^ =.09. The difference in correct responses between the deceptive and truthful condition was greater in the first block, *F*(1, 113) = 93.49, *p* <.001, η_*p*_^2^ =.45, than in the repeated block, *F*(1, 113) = 84.28, *p* <.001, η_*p*_^2^ =.43. Furthermore, a significant Response × Plan interaction occurred, *F*(1, 113) = 25.70, *p* <.001, η_*p*_^2^ =.19. The difference in correct responses between the truthful and deceptive condition block was greater for unplanned than for planned behavior; unplanned behavior: *M*_*truthful*_ = 95.90%, *SE*_*truthful*_ = 0.37, *M*_*deceptive*_ = 89.51%, *SE*_*deceptive*_ = 0.73,* F*(1, 113) = 97.90, *p* <.001, η_*p*_^2^ =.46; planned behavior: *M*_*truthful*_ = 96.18%, *SE*_*truthful*_ = 0.36, *M*_*deceptive*_ = 93.09%, *SE*_*deceptive*_ = 0.55,* F*(1, 113) = 51.51, *p* <.001, η_*p*_^2^ =.31.

**Table 1:** Mean (standard error) accuracies for truthful responses and lies as a function of task repetition

	Truthful	Lie
Block 1	96.15% (0.33)	90.58% (0.65)
Block 2	95.93% (0.31)	92.02% (0.51)

In ANCOVAs with Machiavellianism as a covariate, the Response × Machiavellianism interaction was not significant for the Jones and Paulhus (2014) scale, *F*(1, 112) = 2.12, *p* =.15, η_*p*_^2^ =.02, for the Henning and Six (1977) scale, *F*(1, 112) = 1.27, *p* =.26, η_*p*_^2^ =.01, or for the joint scale, *F*(1, 112) = 0.03, *p* =.86, η_*p*_^2^ =.00. The main effect of Machiavellianism was close to significant for the scale by Henning and Six (1977), *F*(1, 112) = 3.89, *p* =.051, η_*p*_^2^ =.03. On this scale, individuals higher in Machiavellianism tended to respond less accurately during the computer task, *r* =  −.18, *p* =.051. The Machiavellianism main effect was not significant for the Jones and Paulhus (2014) scale, *F*(1, 112) = 0.08, *p* =.78, η_*p*_^2^ =.00, or the joint scale, *F*(1, 112) = 1.54, *p* =.22, η_*p*_^2^ =.01. In the ANOVAs with Machiavellianism as a between-subjects factor, the Response × Machiavellianism interaction was marginally significant for the Jones and Paulhus (2014) scale, *F*(1, 112) = 3.08, *p* =.08, η_*p*_^2^ =.03. The differences in the percentage of correct responses between deceptive and truthful responses tended to be larger for the group of individuals with low Machiavellianism scores, *M*_*truthful*_ = 96.37%, *SE*_*truthful*_ = 0.40, *M*_*deceptive*_ = 90.85%, *SE*_*deceptive*_ = 0.82,* F*(1, 55) = 64.35, *p* <.001, η_*p*_^2^ =.54, than for those with high Machiavellianism scores, *M*_*truthful*_ = 95.72%, *SE*_*truthful*_ = 0.40, *M*_*deceptive*_ = 91.74%, *SE*_*deceptive*_ = 0.71, *F*(1, 57) = 53.02, *p* <.001, η_*p*_^2^ =.48. The Response × Machiavellianism interaction was not significant for the Henning and Six (1977) scale, *F*(1, 112) = 0.32, *p* =.57, η_*p*_^2^ =.00, or the joint scale, *F*(1, 112) = 0.53, *p* =.47, η_*p*_^2^ =.01. The main effect of Machiavellianism was not significant in these analyses for any of the scales (all *p*s >.27).

Truthful responses were 277 ms faster than deceptive responses, *F*(1, 113) = 127.24, *p* <.001, η_*p*_^2^ =.53; *M*_*truthful*_ = 825.84 ms, *SE*_*truthful*_ = 20.56, *M*_*deceptive*_ = 1103.23 ms, *SE*_*deceptive*_ = 37.12. Furthermore, responses for planned behavior were 24 ms faster than for unplanned behavior, *F*(1, 113) = 16.43, *p* <.001, η_*p*_^2^ =.13; *M*_*planned*_ = 952.65 ms, *SE*_*planned*_ = 27.17, *M*_*unplanned*_ = 976.43 ms, *SE*_*unplanned*_ = 27.87. The Response × Plan interaction was not significant, *F*(1, 113) = 2.29, *p* =.13, η_*p*_^2^ =.02. Participants responded 85 ms faster in the repeated block than in the first block of the task, *F*(1, 113) = 62.87, *p* <.001, η_*p*_^2^ =.36; *M*_*first block*_ = 1007.10 ms, *SE*_*first block*_ = 29.79, *M*_*repeated block*_= 921.98 ms, *SE*_*repeated block*_= 25.85. A significant Response × Repetition interaction occurred, *F*(1, 113) = 21.14, *p* <.001, η_*p*_^2^ =.16. The difference in response times between deceptive and truthful responses in relation to *SE* was greater in the repeated block than in the first block of the task (Table 2); first block: *F*(1, 113) = 101.29, *p* <.001, η_*p*_^2^ =.47; repeated block:* F*(1, 113) = 154.25, *p* <.001, η_*p*_^2^ =.58.

**Table 2:** Mean (standard error) response times for truthful responses and lies as a function of task repetition

	Truthful	Lie
Block 1	848.51 ms (20.82)	1165.68 ms (42.88)
Block 2	803.17 ms (21.17)	1040.78 ms (32.73)

In the ANCOVAs with Machiavellianism as a covariate, the Machiavellianism × Response interaction was not significant for the Jones and Paulhus (2014) scale, *F*(1, 112) = 0.46, *p* =.50, η_*p*_^2^ =.00, for the Henning and Six (1977) scale,* F*(1, 112) = 0.23, *p* =.63, η_*p*_^2^ =.00, or the joint scale, *F*(1, 112) = 0.01, *p* =.92, η_*p*_^2^ =.00. The main effect of Machiavellianism was not significant for the Henning and Six (1977) scale or the joint scale (both *p*s >.29), but marginally significant for the Jones and Paulhus (2014) scale, *F*(1, 112) = 3.10, *p* =.08, η_*p*_^2^ =.03. On this scale, individuals with higher Machiavellianism scores tended to respond more slowly, *r* =.16, *p* =.08. In the ANOVAs with Machiavellianism as a between-subjects factor, the Machiavellianism × Response interaction was not significant for any of the three scales: Jones and Paulhus (2014) scale: *F*(1, 112) = 1.34, *p* =.25, η_*p*_^2^ =.01; Henning and Six (1977) scale: *F*(1, 112) = 0.81, *p* =.37, η_*p*_^2^ =.01, joint scale: *F*(1, 112) = 1.15, *p* =.29, η_*p*_^2^ =.01. In these ANOVAs, the main effect of Machiavellianism was also not significant for any of the scales (all *p*s >.15).

### MFN amplitudes

**Fig. 3:**
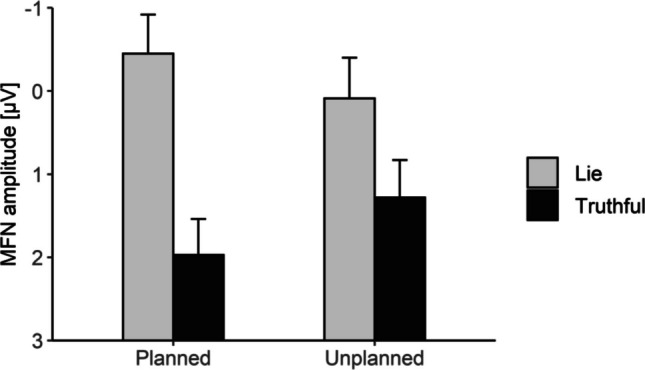
Means and standard errors of MFN amplitudes representing the Response × Plan interaction, i.e., lies and truthful responses about planned and unplanned behavior

As expected, MFN amplitudes were significantly larger for deceptive responses (*M* =  −0.18 µV, *SE* = 0.45) than for truthful responses (*M* = 1.63 µV, *SE* = 0.42; pre-registered hypothesis 1), *F*(1, 113) = 58.47, *p* <.001, η_*p*_^2^ =.34. A significant Response × Plan interaction occurred (Fig. 3)*, F*(1, 113) = 12.42, *p* <.001, η_*p*_^2^ =.10. The difference in MFN amplitudes between deceptive and truthful responses was greater for planned*, F*(1, 113) = 58.37, *p* <.001, η_*p*_^2^ =.34, compared with unplanned behaviors, *F*(1, 113) = 19.63, *p* <.001, η_*p*_^2^ =.15. The main effect for Electrode Position was significant, *F*(3, 339) = 80.22, ε =.52, *p* <.001, η_*p*_^2^ =.42. Simple contrasts revealed that MFN amplitudes were more negative at Fz (*M* =  −0.68 µV, *SE* = 0.41) than at FC1 (*M* = 0.14 µV, *SE* = 0.45, *F*(1, 113) = 21.85, *p* <.001, η_*p*_^2^ =.16), FC2 (*M* = 0.86 µV, *SE* = 0.41, *F*(1, 113) = 84.59, *p* <.001, η_*p*_^2^ =.43), and Cz (*M* = 2.57 µV, *SE* = 0.49, *F*(1, 113) = 95.42, *p* <.001, η_*p*_^2^ =.46). Furthermore, MFN amplitudes were significantly more negative in the first block of the task (*M* =  −0.12 µV, *SE* = 0.42) than in the repeated block (*M* = 1.57 µV, *SE* = 0.44), *F*(1, 113) = 66.97, *p* <.001, η_*p*_^2^ =.37. The main effect Plan as well as the interaction Response × Repetition were both not significant, both *p*s >.69. Likewise, the interaction Electrode Position × Response was not significant, *F*(3, 339) = 2.65, ε =.48, *p* =.09, η_*p*_^2^ =.02.

ANCOVAs with Machiavellianism as a covariate yielded no significant Machiavellianism × Response interaction; neither for the Machiavellianism scale by Jones and Paulhus (2014), *F*(1, 112) = 1.74, *p* =.19, η_*p*_^2^ =.02, nor for the scale by Henning and Six (1977), *F*(1, 112) = 2.93, *p* =.09, η_*p*_^2^ =.03, nor by the joint scale, *F*(1, 112) = 2.85, *p* =.09, η_*p*_^2^ =.03. The main effect Machiavellianism was not significant for any of the three scales (all *p*s >.88). When treating Machiavellianism as a between-subjects factor in the ANOVAs, the Machiavellianism × Response interaction was likewise not significant (scale by Jones & Paulhus, 2014: *F*(1, 112) = 0.20, *p* =.66, η_*p*_^2^ =.00; scale by Henning & Six, 1977: *F*(1, 112) = 0.81, *p* =.37, η_*p*_^2^ =.01; joint scale: *F*(1, 112) = 0.04, *p* =.84, η_*p*_^2^ =.00). In these ANOVAs, the main effect of Machiavellianism was also not significant for any of the three scales (all *p*s >.56).

### PRP amplitudes

**Fig. 4:**
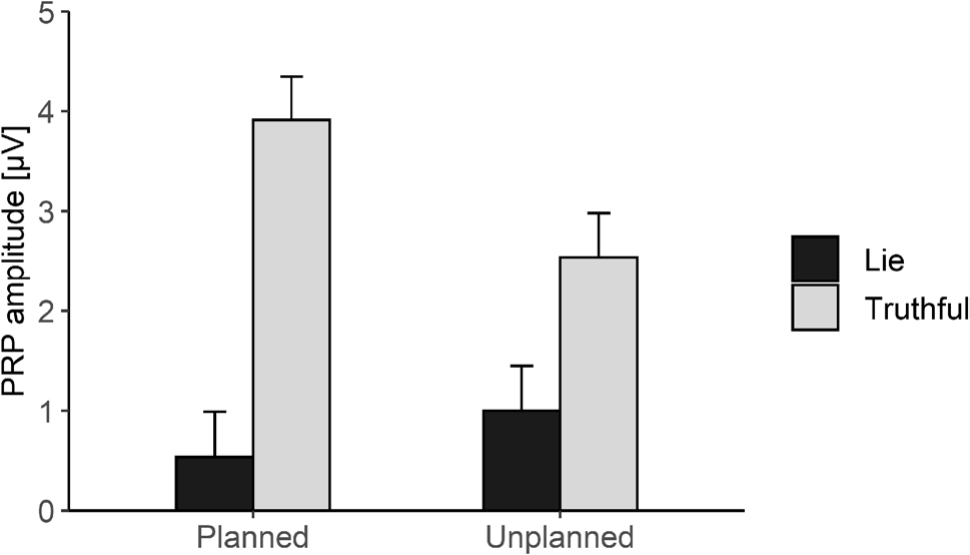
Means and standard errors of PRP amplitudes representing the Response × Plan interaction, i.e., lies and truthful responses about planned and unplanned behavior

As shown in Fig. 2, a positive peak appeared before the response at frontocentral electrodes, depicting the PRP component. PRP amplitudes were significantly less positive for deceptive than truthful responses (*M*_*truthful*_ = 3.23 µV, *SE*_*truthful*_ = 0.42; *M*_*deceptive*_ = 0.77 µV, *SE*_*deceptive*_ = 0.42); *F*(1, 113) = 93.41, *p* <.001, η_*p*_^2^ =.45. Furthermore, PRP amplitudes were significantly less positive for unplanned behavior (*M* = 1.77 µV, *SE* = 0.42) than for planned behavior (*M* = 2.23 µV, *SE* = 0.41), *F*(1, 113) = 4.73, *p* <.05, η_*p*_^2^ =.04. As for MFN amplitudes, a Response × Plan interaction occurred (Fig. 4), *F*(1, 113) = 25.04, *p* <.001, η_*p*_^2^ =.18. The difference in PRP amplitudes between deceptive and truthful responses was greater for planned than for unplanned behavior; planned behavior: *F*(1, 113) = 108.07, *p* <.001, η_*p*_^2^ =.49; unplanned behavior: *F*(1, 113) = 26.49, *p* <.001, η_*p*_^2^ =.19. The main effect Electrode position was significant,* F*(3, 339) = 77.04, ε =.53, *p* <.001, η_*p*_^2^ =.41. Simple contrasts revealed that PRP amplitudes were less positive at Fz (*M* = 0.76 µV, *SE* = 0.40) than at FC1 (*M* = 1.44 µV, *SE* = 0.43, *F*(1, 113) = 17.18, *p* <.001, η_*p*_^2^ =.13), FC2 (*M* = 2.06 µV, *SE* = 0.39, *F*(1, 113) = 70.61, *p* <.001, η_*p*_^2^ =.39), and Cz (*M* = 3.74 µV, *SE* = 0.46, *F*(1, 113) = 91.65, *p* <.001, η_*p*_^2^ =.45). Furthermore, PRP amplitudes were significantly less positive in the first block of the task (*M* = 1.17 µV, *SE* = 0.40) than in the repeated block (*M* = 2.83 µV, *SE* = 0.43), *F*(1, 113) = 68.07, *p* <.001, η_*p*_^2^ =.38. The Electrode Position × Response interaction was significant,* F*(3, 339) = 14.86, ε =.48, *p* <.001, η_*p*_^2^ =.12. Simple contrasts revealed that the difference in PRP amplitudes between deceptive and truthful responses was smaller at Fz (*M*_*truthful*_ = 2.06 µV, *SE*_*truthful*_ = 0.43; *M*_*deceptive*_ = −0.54 µV, *SE*_*deceptive*_ = 0.43) than at FC1 (*M*_*truthful*_ = 2.95 µV, *SE*_*truthful*_ = 0.45; *M*_*deceptive*_ = −0.07 µV, *SE*_*deceptive*_ = 0.47, *F*(1, 113) = 11.56, *p* <.001, η_*p*_^2^ =.09). Furthermore, the difference in PRP amplitudes between deceptive and truthful responses was greater at Fz than at FC2 (*M*_*truthful*_ = 2.70 µV, *SE*_*truthful*_ = 0.43; *M*_*deceptive*_ = 1.42 µV, *SE*_*deceptive*_ = 0.39, *F*(1, 113) = 12.33, *p* <.001, η_*p*_^2^ =.10), but no significant difference occurred between electrode Fz and Cz (*M*_*truthful*_ = 5.21 µV, *SE*_*truthful*_ = 0.48; *M*_*deceptive*_ = 2.27 µV, *SE*_*deceptive*_ = 0.51, *F*(1, 113) = 2.94, *p* =.09, η_*p*_^2^ =.03). The Response × Repetition interaction was not significant, *F*(1, 113) = 20.31, *p* =.45, η_*p*_^2^ =.01.

In the ANCOVAs with Machiavellianism as a covariate, the Machiavellianism × Response interaction was marginally significant for the scale by Jones and Paulhus (2014), *F*(1, 112) = 3.25, *p* =.07, η_*p*_^2^ =.03, and for the joint Machiavellianism scale, *F*(1, 112) = 3.27, *p* =.07, η_*p*_^2^ =.03. To further analyze this interaction, we correlated the differences in PRP amplitudes between deceptive and truthful responses with Machiavellianism scores. A significant correlation of *r* =  −.21 (*p* <.05) for the scale by Jones and Paulhus (2014) at electrode Fz indicated that individuals with lower Machiavellianism scores showed greater differences in PRP amplitudes between deceptive and truthful responses. The correlation was not significant at the other analyzed frontocentral electrodes (all *ps* >.07). For the joint scale, the correlation at electrode Fz was marginally significant, *r* =  −.18, *p* =.06, but nonsignificant at the other frontocentral electrodes (all *ps* >.11). The Machiavellianism × Response interaction was not significant for the scale by Henning and Six (1977), *F*(1, 112) = 2.07, *p* =.15, η_*p*_^2^ =.02. The Machiavellianism main effect was not significant in any of the scales (all *p*s >.60). In the ANOVAs with Machiavellianism as a between-subjects factor, there was no significant Machiavellianism × Response interaction for the scale by Jones and Paulhus (2014), *F*(1, 112) = 0.32, *p* =.57, η_*p*_^2^ =.00, for the scale by Henning and Six (1977), *F*(1, 112) = 1.57, *p* =.21, η_*p*_^2^ =.01, or for the joint scale, *F*(1, 112) = 0.00, *p* =.96, η_*p*_^2^ =.00. The Machiavellianism main effect was also not significant in any of the scales (all *p*s >.20).

### LPC amplitudes

**Fig. 5:**
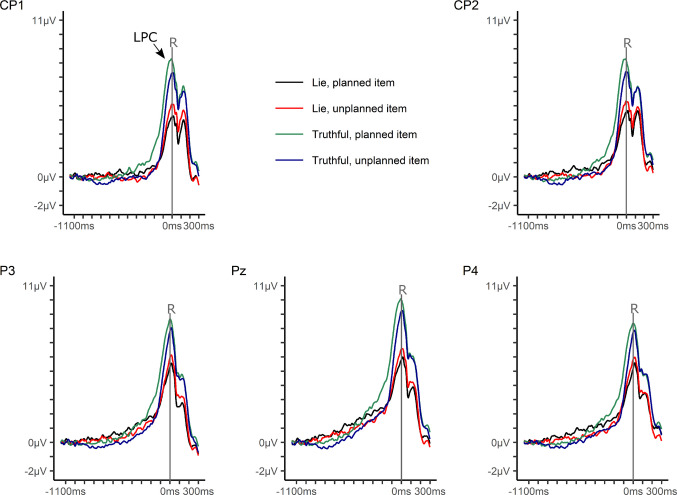
Response-locked grand averages of parietal-central electrodes for lies as well as truthful responses for planned behavior and unplanned behavior. Epochs spanned from 1150 ms before until 300 ms after the response. One tick at the x-axis stands for 100 ms and one tick at the y-axis for 1 µV

**Fig. 6:**
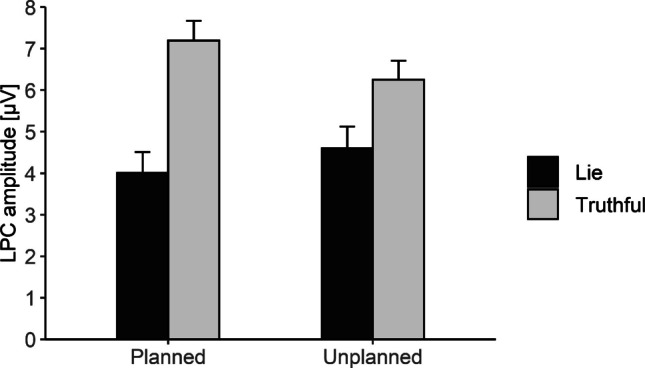
Means and standard errors of LPC amplitudes representing the Response × Plan interaction, i.e., lies and truthful responses about planned and unplanned behavior

As expected, LPC amplitudes were significantly suppressed for deceptive responses (Fig. 5; *M* = 4.30 µV, *SE* = 0.49) compared with truthful responses (*M* = 6.72 µV, *SE* = 0.45; pre-registered hypothesis 2), *F*(1, 113) = 54.29, *p* <.001, η_*p*_^2^ =.33. A significant Response × Plan interaction occurred, *F*(1, 113) = 17.25, *p* <.001, η_*p*_^2^ =.13. The difference in LPC amplitudes between deceptive and truthful responses was greater for planned than for unplanned behavior (Fig. 6), planned behavior: *F*(1, 113) = 67.39, *p* <.001, η_*p*_^2^ =.37; unplanned behavior: *F*(1, 113) = 20.70, *p* <.001, η_*p*_^2^ =.16. Furthermore, the main effect Electrode Position was significant, *F*(4, 452) = 23.03, ε =.78*, p* <.001, η_*p*_^2^ =.17. Simple contrasts revealed that LPCs were larger at Pz (*M* = 6.44 µV, *SE* = 0.50) than at P3 (*M* = 5.37 µV, *SE* = 0.43, *F*(1, 113) = 49.39, *p* <.001, η_*p*_^2^ =.30), P4 (*M* = 5.40 µV, *SE* = 0.42, *F*(1, 113) = 44.57, *p* <.001, η_*p*_^2^ =.28), CP1 (*M* = 5.06 µV, *SE* = 0.46, *F*(1, 113) = 89.78, *p* <.001, η_*p*_^2^ =.44), and CP2 (*M* = 5.29 µV, *SE* = 0.44, *F*(1, 113) = 58.35, *p* <.001, η_*p*_^2^ =.34). LPCs were significantly larger in the repeated block of the task (*M* = 6.83 µV, *SE* = 0.49) than in the first one (*M* = 4.20 µV, *SE* = 0.43, *F*(1, 113) = 99.75, *p* <.001, η_*p*_^2^ =.47). The Electrode Position × Response interaction was significant, *F*(4, 452) = 15.43, ε =.78*, p* <.001, η_*p*_^2^ =.12. Simple contrasts revealed that the difference in LPC amplitudes between deceptive and truthful responses was greater at Pz (*M*_*truthful*_ = 7.89 µV, *SE*_*truthful*_ = 0.51; *M*_*deceptive*_ = 5.00 µV, *SE*_*deceptive*_ = 0.55) than at P3 (*M*_*truthful*_ = 6.43 µV, *SE*_*truthful*_ = 0.45; *M*_*deceptive*_ = 4.30 µV, *SE*_*deceptive*_ = 0.47; *F*(1, 113) = 37.17, *p* <.001, η_*p*_^2^ =.25), P4 (*M*_*truthful*_ = 6.41 µV, *SE*_*truthful*_ = 0.44; *M*_*deceptive*_ = 4.39 µV, *SE*_*deceptive*_ = 0.47; *F*(1, 113) = 30.22, *p* <.001, η_*p*_^2^ =.21), CP1 (*M*_*truthful*_ = 6.38 µV, *SE*_*truthful*_ = 0.47; *M*_*deceptive*_ = 3.74 µV, *SE*_*deceptive*_ = 0.52; *F*(1, 113) = 5.12, *p* <.05, η_*p*_^2^ =.04), and CP2 (*M*_*truthful*_ = 6.50 µV, *SE*_*truthful*_ = 0.46; *M*_*deceptive*_ = 4.09 µV, *SE*_*deceptive*_ = 0.49; *F*(1, 113) = 26.99, *p* <.001, η_*p*_^2^ =.19). The main effect Plan and the interaction Response × Repetition were both not significant, *p*s >.39.

In the ANCOVAs with Machiavellianism as a covariate, the Machiavellianism × Response interaction was not significant for the Jones and Paulhus (2014) scale, *F*(1, 112) = 1.26, *p* =.27, η_*p*_^2^ =.01, the Henning and Six (1977) scale, *F*(1, 112) = 1.73, *p* =.19, η_*p*_^2^ =.02, or the joint scale, *F*(1, 112) = 1.84, *p* =.18, η_*p*_^2^ =.02. Likewise, the Machiavellianism main effect was not significant for any of the scales (all *p*s >.47). The same results were obtained when considering the median-split of Machiavellianism as a between-subjects factor: The Machiavellianism × Response interaction was not significant for any of the three scales (all *p*s >.43), nor was the main effect of Machiavellianism (all *p*s >.56).

## Discussion

The present study investigated cognitive processes during deception about planned behavior by analyzing brain and behavioral data. Thereby, deception about everyday topics (e.g., planning to get married, graduating, going to a concert) was investigated in a controlled laboratory study. We aimed to extend previous neurocognitive findings of deception about attitudes (Johnson et al., 2008; Scheuble & Beauducel, 2020a) to a more specific behavioral correlate. Examining deception about planned behavior provides a conceptual link between studies on attitudes and those in forensic contexts or applying the CIT, which typically involve deception related to specific behaviors (e.g., theft; Rosenfeld, 2020; Suchotzki et al., 2015). We analyzed a temporal dimension that is scarcely investigated in deception research—namely behavior that is likely to occur in the future but does not necessarily have to. We expect that individuals keep their plans in mind until they can perform the intended behavior in the future. Therefore, no retrieval of previously learned material is necessary. The stimuli of the task were individually tailored to each participant in order to represent strongly held planned/unplanned behavior. Consistent with the TPB, planned behavior was also conceptualized as wanted behavior and unplanned behavior as unwanted behavior (Ajzen, 1991; Fini et al., 2012; Guraya et al., 2024; Hsu & Huang, 2012; Sheeran et al., 2005). Responses were significantly less accurate and slower for deceptive compared to truthful responses. These behavioral results are in line with previous studies and support the assumption that deception entails greater cognitive demands (Johnson et al., 2008; Scheuble & Beauducel, 2020a, 2020b; Suchotzki et al., 2015, 2017). MFN amplitudes were significantly more negative for deceptive than truthful responses (confirmed pre-registered hypothesis 1). Enlarged MFN amplitudes have been associated with response conflicts (Bartholow et al., 2005). Accordingly, deception was accompanied by greater response conflict and relied on response-inhibition processes. Furthermore, for deception about planned behavior, a newly discovered ERP occurred, the PRP. The PRP component was found in previous studies for deception about attitudes and deception requiring strategic monitoring (Johnson et al., 2004, 2005, 2008). In line with those previous studies, PRP amplitudes were significantly suppressed for deceptive compared to truthful responses. Accordingly, deception about planned behavior engaged strategic monitoring processes. Likewise, LPC amplitudes were significantly suppressed for deceptive than truthful responses (confirmed pre-registered hypothesis 2). This LPC pattern aligns with the interpretation that deception constitutes an additional cognitive task, which is more effortful and impedes stimulus processing (Johnson, 1986). Additionally, we investigated whether repetition of the task blocks affected ERP amplitudes. The first half of the task consisted of a deceptive and a truthful condition block, which were subsequently repeated in the second half of the task. For the Repetition factor, ERP amplitudes were compared between the two task halves. It should be noted that within each condition block (i.e., the deceptive and truthful condition blocks), each planned or unplanned behavior stimulus was also presented twice. The additional cognitive processes associated with lying—namely greater response inhibition, strategic monitoring, and higher cognitive demands as indicated by MFN, PRP, and LPC amplitudes—remained stable across repetitions of the task blocks and thus were not affected by practice. Likewise, greater response inhibition and cognitive demands for deception (as indicated by MFN and LPC amplitudes) were stable across individual differences in Machiavellianism. However, a significant valence asymmetry emerged: The patterns of MFN, PRP, and LPC amplitudes indicated that lying about planned behavior was accompanied by greater response conflicts, strategic monitoring, and cognitive demands than lying about unplanned behavior.

The finding that MFN amplitudes were significantly enlarged for deceptive compared with truthful responses is in line with previous deception studies on attitude ratings (Johnson et al., 2008; Scheuble & Beauducel, 2020a), the memorization of stimuli (Johnson et al., 2004, 2005), and studies applying the CIT (Scheuble & Beauducel, 2020b; Scheuble et al., 2021). In these studies, the patterns of MFN amplitudes have been associated with greater response conflict and response inhibition processes for deceptive compared to truthful responses, as lying requires keeping both the truthful and the deceptive response in mind. However, in a study by Suchotzki et al. (2015), a converse pattern was found for MFN amplitudes. In this study, participants planned a mock theft and afterward completed the Sheffield lie task. Participants were motivated through the promise of an additional reward contingent on successful deception. They found, as in the present study, that response times were longer for deceptive than truthful responses and, accordingly, cognitively more demanding. Yet, MFN amplitudes were enlarged for truthful in comparison to deceptive responses. They argued that the MFN component is also sensitive to the emotional valence, and smaller MFN amplitudes may have occurred for deceptive responses because they were rewarded and therefore possibly perceived as the more accurate response. Similarly, in a study by Scheuble and Beauducel (2020b), MFN amplitudes appeared to be sensitive to the moral processing of lying, as the difference in MFN amplitudes between deceptive and truthful responses was attenuated in a condition in which lying could be perceived as a prosocial act intended to help another person. In sum, the present study extends previous deception research in finding that significantly enlarged MFN amplitudes occur for deception about planned behavior in a nonforensic context—an effect associated with stronger response conflicts during deception. Future studies are warranted to determine whether this result remains robust when participants are incentivized to lie successfully.

Furthermore, the present study corroborates the relevance of the PRP component. This newly discovered ERP component in deception tasks, which has been found for deception about attitudes (Johnson et al., 2008; Scheuble & Beauducel, 2020a) and deception requiring strategic monitoring, also occurred in the present study for deception about planned behavior. It is a component preceding the deceptive response and therefore possibly able to predict deception. In line with deception studies on attitudes, we found significantly suppressed PRP amplitudes for deception about a more specific behavioral correlate, namely planned behavior. Because PRP amplitudes have been associated with strategic monitoring, it suggests that not only deception about attitudes but also other types of deception involve strategic monitoring. In the present study, this was the case for planned behavior, that is, behavior that potentially takes place in the future but does not necessarily have to.

The finding of significantly suppressed LPC amplitudes for deceptive compared with truthful responses, suggesting a higher cognitive demand for lies, is in line with previous deception research that did not employ the CIT. These patterns of LPC amplitudes were also found for deception about attitudes (Johnson et al., 2008; Scheuble & Beauducel, 2020a), the memorization of stimuli (Johnson et al., 2003), and deception about a planned mock theft (Suchotzki et al., 2015). Conversely, studies applying the CIT have reported more positive LPC amplitudes for probe items—requiring the concealment of knowledge—than for irrelevant items (Rosenfeld, 2020). This pattern of LPC amplitudes has been associated with a higher salience for probes (Rosenfeld, 2020). The results of the present study therefore revealed that also when lying about a specific, behavior-related construct in a nonforensic context, patterns of LPC amplitudes suggest a higher cognitive demand during deception, as has been found in previous studies that did not employ the CIT.

Furthermore, a significant valence asymmetry was found, with larger differences between deceptive and truthful responses across all ERPs for planned than for unplanned behavior. The MFN amplitudes indicated that greater response conflict was associated with deception about planned behavior compared to deception about unplanned behavior. The pattern of PRP amplitudes suggested greater strategic monitoring for deception about planned compared to unplanned behavior. Likewise, LPC patterns suggested a greater cognitive demand for deception about planned behavior compared to deception about unplanned behavior. Even though we did not state a hypothesis for this pattern, it fits well with previous results regarding attitudes. Former studies analyzing MFN and LPC amplitudes found that lying was cognitively more strenuous and associated with greater response conflict for items rated as positive compared with those rated as negative (Johnson et al., 2008; Scheuble & Beauducel, 2020a). In these studies, a main effect of valence was found for PRP amplitudes but not for the interaction between valence and the response (deceptive vs. truthful). In contrast, in the present study, such an interaction effect was observed, with a greater difference in strategic monitoring between truthful and deceptive responses for planned compared to unplanned behavior. Lying seems to be more cognitively demanding when it concerns items that people are attracted to, compared with those they tend to avoid. Johnson et al. (2008) explained this positive–negative asymmetry that they found for attitudes by suggesting that lying about positively rated items may be perceived as a denial of the self and therefore be accompanied by more conflicts. Conversely, lying about negatively rated items may be perceived as a sort of compliance, which is more common (Johnson et al., 2008). This explanation could also account for our results for plans: Plans have been closely related to self-identity (Carver et al., 1999; Higgins, 1987; Sheldon et al., 1999). Because many studies have found an association between self-identity and planned behavior, it has been argued that self-identity should be integrated as a predictor in the TPB (Rise et al., 2010; Smith et al., 2007; Sparks & Guthrie, 1998). In this line of research, it has been suggested that planned behavior is motivated by the desire to reinforce, support, and confirm one’s self-identity and that the self guides the formation of behavior plans (Rise et al., 2010; Stets & Burke, 2000). Conversely, “[g]oals that are not endorsed by the self are likely to generate intrapersonal conflict […]” (Koestner et al., 2002, p. 231). Plans that are not self-concordant can be distressing (Sheldon & Elliot, 1999). Our finding of a valence asymmetry for deception about planned vs. unplanned behavior could therefore be explained as follows. When a behavior is planned, individuals may hold a strong position toward this behavior, which is possibly also intertwined with their self-identity. Because of this strong position towards planned behavior, lying about it may be more difficult and accompanied by greater conflict and cognitive effort. Conversely, people might not place much weight on what they say about unplanned behavior. They may be less attached to unplanned behavior and more willing to deceptively claim that they engage in it, even if they do not plan to do so. Therefore, they possibly care less about lying about unplanned behavior, which could explain why it was accompanied by less conflict and lower cognitive demand. In the present study, the planned and unplanned behaviors were individually selected for each participant based on their ratings of the extent to which they plan to perform the behavior (with a response scale ranging from absolutely unwanted to absolutely wanted) and based on their ratings of how likely they were to maintain their plans. Even though both planned and unplanned behaviors were rated as highly likely to be maintained, with mean ratings of at least approximately one point below the maximum of the 7-point response scale (1 = absolutely unlikely, 7 = absolutely likely), participants reported a higher likelihood of maintaining their planned behaviors. Valence asymmetries could also be found in further previous research studies. Likewise, it is harder for people to reconsider negative features of items they like than to reconsider positive features for negatively rated items (Gershoff et al., 2008). Moreover, they are more likely to overestimate the extent to which others agree with their positive ratings compared with their negative ratings (Gershoff et al., 2008). People also lie more frequently about negative topics (DePaulo et al., 1996), i.e., by pretending something is positive when they actually consider it negative. These results are in line with our finding that it is cognitively more demanding to lie about planned behavior, which is likely to be more positively evaluated than unplanned behavior that may have more negative connotations. However, our conclusions remain tentative pending replication. In particular, the proposed possible explanation regarding the self-relevance of individually planned behavior needs to be examined further by incorporating self-importance ratings of the deception-task items, which were not collected in the present study. Future studies are needed to more thoroughly investigate possible explanations for the observed valence asymmetries in cognitive effort during deception about planned vs. unplanned behavior.

Additionally, we explored moderation effects of Machiavellianism on the cognitive processes of deception about planned behavior. To more thoroughly investigate these effects, we employed multiple Machiavellianism scales and conducted two different statistical analyses, treating Machiavellianism either as a covariate or as a between-subjects factor in the ANOVAs. The effects of MFN amplitudes remained stable across individual differences in Machiavellianism, regardless of the statistical analyses used. Accordingly, in our study, individuals higher in Machiavellianism did not experience fewer response conflicts during deception. However, it should be noted that the Machiavellianism x Response interaction was marginally significant for the MFN and the scale of Henning and Six (1977). Accordingly, in future studies on Machiavellianism and deception, it seems important to consider this scale and further examine whether a significant effect emerges in other deception contexts or with a larger sample size. Machiavellianism did not moderate the effects on LPC amplitudes, revealing that deception was not cognitively less demanding for individuals higher in Machiavellianism. The MFN and LPC amplitudes appear to be stable markers of deception across individual differences in Machiavellianism. This fits well with Scheuble and Beauducel (2020a) investigating lies about attitudes: Likewise, no moderation effect of Machiavellianism was found for the cognitive processes during lies indicated by MFN and LPC amplitudes. At least in the deception paradigms on attitudes and plans investigated until now, it appears that MFN and LPC components are primarily influenced by the required cognitive resources rather than by the individual’s moral processing. However, for PRP amplitudes, a greater difference between deceptive and truthful responses tended to emerge for individuals with lower Machiavellianism scores on the scale by Jones and Paulhus (2014) and the joint Machiavellianism (combining the scales by Jones & Paulhus, 2014 and Henning & Six, 1977), when these scores were treated as covariates. Accordingly, deception tended to be accompanied by greater strategic monitoring for individuals lower in Machiavellianism. However, the described results need to be interpreted with extra caution, because they were only marginally significant and occurred only for some scales of Machiavellianism. In contrast, in the previous deception study on attitudes, moderating effects of Machiavellianism on PRP amplitudes were not significant. In sum, for lies about planned behavior, individuals lower in Machiavellianism tended to experience greater strategic monitoring, whereas they did not experience greater response conflicts or increased cognitive demands. Machiavellianism may have been irrelevant for the cognitive load and response conflicts because lying or telling the truth did not result in any advantage or disadvantage in the present paradigm; thus, the task did not constitute a high-stakes deception context. According to Jones and Mueller (2021), it is important to clarify under which circumstances Machiavellianism is associated with unethical behavior rather than whether it predicts unethical behaviors at all. In line with this perspective, it remains important to further examine the moderating role of Machiavellianism across different deception contexts. Nevertheless, the present results underline the similarity of the cognitive processes during lying across individuals with varying levels of Machiavellianism in the present paradigm. Even though previous research has found that individuals higher in Machiavellianism report having greater deception abilities (Gozna et al., 2001; Wissing & Reinhard, 2019), our findings suggest that higher Machiavellianism is not associated with reduced cognitive demands or response conflicts during deception about planned behavior per se.

Notably, no significant difference occurred in the pattern of MFN, PRP, and LPC amplitudes when the task blocks were repeated. Accordingly, deception was not accompanied by reduced response conflict, strategic monitoring, or cognitive effort for the repetition of the task, and no significant practice effects occurred. Deception about planned behavior does not seem to become cognitively easier with practice. This suggests that, for individually planned behavior, the analyzed ERP components are stable markers of deception, even in longer deception tasks. Hence, task repetition seems to be a suitable approach for improving the signal-to-noise ratio by averaging ERPs over more trials for deception about planned behavior. Conversely, in a study by Johnson et al. (2005) investigating deception about the recognition of words, practice effects emerged for MFN, PRP, and LPC components. They found practice-related changes in each of these components for truthful but not for deceptive responses. Accordingly, with practice, fewer executive processes were required for truthful responses. The different results for the two deception tasks may be explained as follows: In the recognition task by Johnson et al. (2005), participants learned the relevant words one week prior to their participation in the study. Conversely, in the present study the stimuli of the task fitted to the individually planned behaviors, which the participants already had in their minds prior to the study. It is possible that the individually planned behaviors were present in the long-term memory of the participants long before. Therefore, truthful responses may be more resistant to practice effects for stimuli that are individually more important and more easily accessed from long-term memory. The resistance of ERP components to practice effects in our deception task on planned behavior aligns with our finding that lying was not cognitively less demanding for individuals higher in Machiavellianism, that is, those who also tend to lie more frequently. In sum, it underlines the stability of the ERP patterns. Yet, it has to be noted that additional research is warranted for a further interpretation of practice effects on cognitive processes during lying.

### Limitations and future directions

Participants had medium scores on the Machiavellianism scales, comparable to those of previous studies (Jones & Paulhus, 2014; Rauthmann, 2012). However, it is possible that mediation effects of Machiavellianism on the cognitive processes involved in lying might emerge in samples with more extreme values. On a related note, it has to be considered that participants received no reward for lying successfully. It remains for future studies to determine whether incentives are necessary to detect moderating effects of Machiavellianism. We aimed at recruiting participants that are not predominantly psychology students. Nevertheless, nearly half of the sample consisted of psychology students, a group that is frequently studied. It remains an open question whether our results can be generalized to more diverse samples with only a minimal proportion of psychology students. In particular, it would be of interest whether the non-significant results for Machiavellianism would also be observed in a sample without psychology students.

Indicating one’s own plans is probably a quick and easy decision. Therefore, it is questionable whether the same ERP patterns—associated with increased cognitive effort and conflict during lying—can also be observed for more complex decisions. More complex decisions may involve greater strategic and moral judgments. Thus, further research is necessary, focusing on complex decisions. Furthermore, it should be noted that lies were planned beforehand by instructing participants. Even though some lies in everyday life are also strategically planned beforehand, this does not apply to spontaneous lies. It is possible that different ERP patterns occur for these (Johnson et al., 2005). Therefore, an intriguing question for future research is which ERP patterns and cognitive processes occur for spontaneous lies about planned behavior.

On a related note, it should be acknowledged as a limitation that we conducted a controlled laboratory study, which reduces its ecological validity. The design was chosen, because the present study represents one of the first to analyze deception related to nonforensic planned behavior and to allow better comparison with prior laboratory deception studies. Participants lied about everyday topics in the form of their individually planned behavior. By focusing on this type of deception, we aimed to take a further step toward capturing individually planned everyday behavior. Nevertheless, important aspects of everyday deception were not represented in our study. The deception had no real-world consequences and remained a low-risk laboratory task. Furthermore, there was no interpersonal contact during the act of deception. Therefore, future studies are needed to investigate deception about planned behavior also in everyday life, for instance, using ambulatory EEG.

The deception task was in line with previous studies (Johnson et al., 2003, 2005, 2008; Scheuble & Beauducel, 2020a). However, a limitation of the present study is that it focused exclusively on the cognitive aspects of deception, whereas motivational and emotional aspects were not manipulated. In the study by Suchotzki et al. (2015), in which converse patterns of MFN amplitudes were observed, the Sheffield lie task was employed, and participants were motivated through the promise of an additional reward for successful deception. They discussed that rewarding participants could be a possible explanation for the MFN patterns, as “lie responses may have been perceived as the actual ‘correct’ response” (Suchotzki et al., 2015, p. 402). Accordingly, rewarding or penalizing deception could have an effect on how deception is perceived by participants. Although reinforcement conditions are probably an important moderator of the MFN in deception contexts, we preferred to start the investigation of deception about planned behavior in a neutral context. Nevertheless, as a next step, it seems crucial for future research to systematically investigate the moderating effects of incentives and punishments on deception about planned behavior. Furthermore, future research is needed that systematically compares the different cognitive, motivational, and emotional aspects of deception, manipulating each independently. In particular, future studies could manipulate deception independently of cognitive load. Moreover, deception can be operationalized in multiple ways, each of which is embodied in different task paradigms. In the present deception task, participants were instructed before each task block to respond either deceptively or truthfully. Accordingly, in the deceptive condition, participants were required to respond deceptively repeatedly across multiple trials, with the exception of catch trials, for which truthful responses were required. Although this operationalization is consistent with previous research (Dionisio et al., 2001; Hu et al., 2011, 2012; Johnson et al., 2008; Scheuble & Beauducel, 2020a, 2020b), it differs from deception paradigms in which the instruction to lie is given as a cue before each trial, such as in the Sheffield lie task. In the present paradigm, participants therefore had to keep in mind to respond deceptively (or truthfully) across the course of the respective task block. Likewise, in conversations, there are potentially different progressions of deception: In some cases, individuals may prepare themselves to respond deceptively on certain topics and maintain deceptive responding over an extended period (Garrett et al., 2016; O’Connor et al., 2020; Reeck & Ariely, 2025), whereas in other contexts they may frequently switch between deceptive and truthful responses. It remains to be investigated whether the present findings hold true when participants equally switch between deceptive and truthful responses. Direct comparisons between paradigms—such as the Sheffield lie task and the deception paradigm employed in the present study as well as in Johnson et al., (2003, 2005, 2008)—are warranted to elucidate task-specific ERP patterns and to resolve the opposing neural patterns reported across different deception studies.

## Conclusions

This is one of the first studies to shed light on lies about individual plans concerning one’s own future life, thereby addressing deception about plans related to everyday behavior. Differential patterns in response times and ERP components between deceptive and truthful responses revealed that deception relied on additional executive processes. The differences between various ERP components elicited by truthful and deceptive responses indicate that deception was accompanied by greater response conflicts, strategic monitoring, and it required additional mental workload compared with truthful responding. Moreover, plans refer to individual future behavior and should therefore be relevant for the individual. Therefore, the present study corroborates the relevance of inhibition of planned behavior as a process during deception that might increase cognitive load. The increased cognitive load was indicated by slower responses, enlarged MFN amplitudes, and suppressed PRP as well as LPC amplitudes for deception. Furthermore, a valence asymmetry appears to exist between planned and unplanned behavior; deception about planned behavior was associated with greater response conflict, increased strategic monitoring, and higher cognitive demands. The differences between deceptive and truthful responses in MFN, PRP, and LPC amplitudes were larger for planned compared to unplanned behavior. Individuals with lower Machiavellianism scores did not experience greater response conflict or cognitive demand during deception, as indicated by stable MFN and LPC amplitude patterns across individuals. An effect of Machiavellianism on the pattern of PRP amplitudes—suggesting that deception was accompanied by greater strategic monitoring for individuals lower in Machiavellianism—could not be ruled out, because some of the corresponding analyses were marginally significant. Repeated acts of deception were likewise associated with increased response conflict, enhanced strategic monitoring, and higher cognitive demands. Practice did not affect the effects of deception on MFN, PRP, and LPC amplitudes, suggesting that deception tasks on planned behavior can include many deceptive and truthful trials in order to improve the signal-to-noise ratio in the analyzed ERPs. In sum, MFN, PRP, and LPC amplitudes seem to be stable markers for deception about plans. The results encourage future ERP studies on lying beyond the recognition context and beyond the forensic context, including lies about future behavior—an area still rarely investigated.

## Supplementary Information

Below is the link to the electronic supplementary material.

Supplementary file1 (DOCX 35 KB)

## Data Availability

Data will be made available upon request. The study was preregistered at osf (https://osf.io/6syqj/?view_only=cde80f6b7f494791ade8725444da6fb4). The preregistration adheres to the disclosure requirements of the institutional registry.

## Code availability

Code will be made available upon request.
