# Supplementary material for: Inhibition and cognitive workload during deception about planned behavior: Investigating LPC, MFN, and PRP components
Source: Cogn Affect Behav Neurosci. 2026 Mar 18;26(4):1577–96. doi: 10.3758/s13415-026-01430-4 (PMC13385121; doi:10.3758/s13415-026-01430-4)
Supplement: Supplementary file 1 — Supplementary file1 (DOCX 35 KB) [file 13415_2026_1430_MOESM1_ESM.docx]

**Supplementary Material**

Inhibition and cognitive workload during deception about planned behavior:

Investigating LPC, MFN, and PRP components

| Items from the questionnaire about individual plans in German | English translation |
| --- | --- |
| HOCHZEIT | MARRIAGE |
| KINDER HABEN | HAVING CHILDREN |
| ABSCHLUSS STUDIUM | GRADUATING FROM UNIVERSITY |
| ABSCHLUSS AUSBILDUNG | APPRENTICESHIP COMPLETION |
| THERAPEUTENAUSBILDUNG | PSYCHOTHERAPY TRAINING |
| SANITÄTERAUSBILDUNG | PARAMEDIC TRAINING |
| GROßE REISE | BIG TRIP |
| WETTKÄMPFE | COMPETITIONS |
| MUSKELAUFBAU | MUSCLE BUILDING |
| ABNEHMEN | LOSING WEIGHT |
| BEFÖRDERUNG | PROMOTION |
| TEILZEITJOB | PART-TIME JOB |
| WORK-LIFE-BALANCE | WORK-LIFE BALANCE |
| HAUS | HOUSE |
| AUSWANDERN | MOVING ABROAD |
| HAUSTIER | PET |
| FORTBILDUNG | TRAINING COURSE |
| BEZIEHUNGEN VERTIEFEN | DEEPENING RELATIONSHIPS |
| NETWORKING | NETWORKING |
| BEKANNTENKREIS ERWEITERN | MEETING NEW PEOPLE |
| EHRENAMT | VOLUNTEERING |
| NACHHALTIGKEIT | SUSTAINABILITY |
| ENERGIE SPAREN | SAVING ENERGY |
| GELD SPAREN | SAVING MONEY |
| DANKBARKEIT | GRATITUDE |
| ANDEREN HELFEN | HELPING OTHERS |
| HOME OFFICE | REMOTE WORK |
| UMZUG | MOVING |
| POSITIV DENKEN | POSITIVE THINKING |
| ACHTSAMKEIT | MINDFULNESS |
| GEDULD | PATIENCE |
| MOTORRAD | MOTORCYCLE |
| GESUNDE ERNÄHRUNG | HEALTHY EATING |
| NÄHKURS | SEWING CLASS |
| SABBATICAL | SABBATICAL |
| BERGSTEIGEN | MOUNTAINEERING |
| STRANDURLAUB | BEACH HOLIDAY |
| STÄDTEREISE | CITY TRIP |
| SKI FAHREN | SKIING |
| PROGRAMMIEREN LERNEN | LEARNING CODING |
| BUNDESWEHR | GERMAN ARMED FORCES |
| GLATZE RASIEREN | HEAD SHAVE |
| STEUER MACHEN | DOING TAXES |
| GEBURTSTAG FEIERN | CELEBRATING BIRTHDAY |
| FLEISCH ESSEN | EATING MEAT |
| PADDELTOUR | PADDLING TOUR |
| YOGA AUSBILDUNG | YOGA TEACHER TRAINING |
| WEINWANDERUNG | WINE TOUR |
| REGIONAL EINKAUFEN | BUY LOCAL |
| DEUTSCHLANDURLAUB | HOLIDAY IN GERMANY |
| TRENNUNG | BREAKUP |
| PLAYSTATION KAUFEN | BUYING A PLAYSTATION |
| PRAKTIKUM | INTERNSHIP |
| JOBSUCHE | JOB SEARCH |
| AUTO | CAR |
| STUDIENFACHWECHSEL | SWITCHING MAJORS |
| AUSLANDSJAHR | YEAR ABROAD |
| KLASSENTREFFEN | CLASS REUNION |
| SPRACHKURS | LANGUAGE COURSE |
| RENOVIEREN | RENOVATING |
| VEGAN | VEGAN |
| KÜNDIGUNG | RESIGNING |
| GESCHLECHTSWECHSEL | GENDER TRANSITION |
| NEUES IPHONE | NEW IPHONE |
| RELIGIONSWECHSEL | RELIGIOUS CONVERSION |
| AFFÄRE | AFFAIR |
| TÄTOWIERUNG | GETTING A TATTOO |
| NAMENSWECHSEL | NAME CHANGE |
| PROMOTION | JOB PROMOTION |
| BUCH SCHREIBEN | BOOK WRITING |
| LIED SCHREIBEN | SONGWRITING |
| DATING | DATING |
| KIRCHENAUSTRITT | LEAVING CHURCH |
| PIERCING | PIERCING |
| GESANGSUNTERRICHT | SINGING LESSONS |
| NEUE SPORTART | NEW SPORT |
| FÜHRERSCHEIN | DRIVING LICENSE |
| JAGDSCHEIN | HUNTING LICENSE |
| TANZKURS | DANCE CLASS |
| INSTRUMENT LERNEN | LEARNING AN INSTRUMENT |
| WELTREISE | WORLD TOUR |
| KREUZFAHRT | CRUISE |
| FLUGSCHEIN | PILOT LICENSE |
| THERAPIE | THERAPY |
| FITNESSSTUDIO | GYM |
| MARATHON | MARATHON |
| ROADTRIP | ROAD TRIP |
| FLUGREISE | AIR TRAVEL |
| SCHLAFUMSTELLUNG | CHANGING SLEEP PATTERNS |
| CAMPINGURLAUB | CAMPING TRIP |
| KONZERTBESUCH | GOING TO A CONCERT |
| SCHÖNHEITS-OP | PLASTIC SURGERY |
| FASTEN | FASTING |
| DROGEN AUSPROBIEREN | TRYING DRUGS |
| KARNEVAL FEIERN | CELEBRATING CARNIVAL |
| KIRCHLICH HEIRATEN | GETTING MARRIED IN CHURCH |
| GEHALTSERHÖHUNG | PAY RAISE |
| ZUSAMMEN ZIEHEN | MOVING IN TOGETHER |
| KLOSTEREINTRITT | JOINING A MONASTERY |
| UNTERNEHMEN GRÜNDEN | STARTING A BUSINESS |
| FALLSCHIRM SPRINGEN | SKYDIVING |
| TAUCHKURS | DIVING COURSE |
| PARTEIEINTRITT | JOINING A POLITICAL PARTY |
| STIPENDIUM | SCHOLARSHIP |
| BAND GRÜNDEN | FORMING A BAND |
| BLUTSPENDE | BLOOD DONATION |
| ORGANSPENDE | ORGAN DONATION |
| EHEVERTRAG | PRENUP |
| VORRAT ANLEGEN | STOCKING UP |
| SELBSTVERTEIDIGUNGSKURS | SELF-DEFENSE COURSE |
| URLAUB ALLEINE | SOLO HOLIDAY |
| POLARLICHTER SEHEN | SEE THE NORTHERN LIGHTS |
| COMEDY-KARRIERE | COMEDY CAREER |
| GLÜCKSSPIEL | GAMBLING |
| AHNENFORSCHUNG | GENEALOGY |
| BILDUNG | EDUCATION |
| GELASSENHEIT | COMPOSURE |
| STROMANBIETER WECHSELN | SWITCHING ELECTRICITY PROVIDERS |
| FESTIVALBESUCH | GOING TO A FESTIVAL |
| KOCHKURS | COOKING CLASS |
| IMPFUNG | VACCINATION |
| VAN KAUFEN | BUYING A VAN |
| BOTOX-BEHANDLUNG | BOTOX TREATMENT |
| MEHR LERNEN | LEARNING MORE |
| REICHTUM | WEALTH |
| FÜHRUNGSPOSITION | LEADERSHIP POSITION |
| GUTE NOTEN | GOOD GRADES |
| SURFEN LERNEN | LEARNING TO SURF |
| FAMILIENGRÜNDUNG | STARTING A FAMILY |
| PASSIVES EINKOMMEN | PASSIVE INCOME |
| ELTERNZEIT | PARENTAL LEAVE |
| BOOT KAUFEN | BUYING A BOAT |
| ROLEX KAUFEN | ROLEX PURCHASE |
| PELZ KAUFEN | BUYING FUR |
| SOLARANLAGE | SOLAR PANELS |
| EIGENTUMSWOHNUNG | CONDOMINIUM |
| FRÜHRENTE | EARLY RETIREMENT |
| BIBELKREIS BEITRETEN | JOINING A BIBLE GROUP |
| FINANZIELLE UNABHÄNGIGKEIT | FINANCIAL INDEPENDENCE |
| ALLEIN SEIN | BEING ALONE |
| ARTIKEL SCHREIBEN | WRITING AN ARTICLE |
| HAARE FÄRBEN | DYEING MY HAIR |
| DIGITAL DETOX | DIGITAL DETOX |
| DJ | DJ |
| REDE HALTEN | GIVING A SPEECH |
| NORDKOREA REISE | NORTH KOREA TRIP |
| WG GRÜNDEN | START ROOMSHARING |
| USA REISE | USA TRIP |
| WELTALLFLUG | SPACE TRAVEl |
| KARRIERE | CAREER |
| MEHR SPORT | WORKING OUT MORE |
| E-BIKE KAUFEN | E-BIKE PURCHASE |
| ERFÜLLENDER JOB | FULFILLING JOB |
| FOTOGRAFIE LERNEN | LEARNING PHOTOGRAPHY |
| PILGERREISE | PILGRIMAGE |
| HABILITATION | HABILITATION |
| TEILNAHME KONFERENZ | CONFERENCE ATTENDANCE |
| WEIHNACHTEN FEIERN | CELEBRATING CHRISTMAS |
| ENGLISCH VERBESSERN | IMPROVING ENGLISH |
| SELBSTBEWUSSTSEIN | SELF-CONFIDENCE |
| KIRCHENFREIZEIT | CHURCH RETREAT |
| UMWELTBEWUSSTER LEBEN | LIVE MORE ECO-FRIENDLY |
| FLÜCHTLINGE AUFNEHMEN | HOSTING REFUGEES |
| TEILNAHME FACHSCHAFT | JOINING THE STUDENT COUNCIL |
| MEDITATIONSKURS | MEDITATION COURSE |
| EISBADEN | ICE BATHING |
| IRON MAN | IRONMAN RACE |
| HUNGERSTREIK | HUNGER STRIKE |
